# Transcranial magnetic stimulation measures of corticospinal excitability in Black and Hispanic/Latino people with painful peripheral neuropathy

**DOI:** 10.3389/fnhum.2025.1551931

**Published:** 2025-06-02

**Authors:** Marlon L. Wong, Lisa M. McTeague, Gabriel Gonzalez, Juan P. Gonzalez, Jessica L. Bolanos, Peter J. Hosein, Danylo F. Cabral, Peter J. Fried

**Affiliations:** ^1^Department of Physical Therapy, University of Miami, Coral Gables, FL, United States; ^2^Department of Psychiatry and Behavioral Sciences, Medical University of South Carolina, Charleston, SC, United States; ^3^Harvard Medical School Department of Neurology, Berenson-Allen Center for Noninvasive Brain Stimulation, Boston, ML, United States

**Keywords:** transcranial magnetic stimulation, peripheral neuropathy, diabetic neuropathy, chemotherapy induced peripheral neuropathy, health disparities

## Abstract

**Introduction:**

This study aims to provide preliminary descriptive data on transcranial magnetic stimulation (TMS) measures obtained in Black and Hispanic/Latino individuals with chronic painful peripheral neuropathy (PN), including those with chemotherapy-induced peripheral neuropathy (CIPN) and diabetic neuropathy (DN). Both CIPN and DN share similar neuropathic symptoms and underlying physiological mechanisms, in particular altered central nervous system processing. TMS is a non-invasive technique that can assess corticospinal excitability and the function of GABAergic and glutamatergic pathways, potentially serving as a diagnostic or prognostic tool for PN.

**Methods:**

This study utilized data from a pilot randomized sham-controlled trial that tested the impact of patient education videos on the effect of transcutaneous auricular vagus nerve stimulation (taVNS) in Black and Hispanic/Latino individuals living with PN. TMS measures, including resting motor threshold (RMT), MEP amplitude following unconditioned single-pulse TMS (spTMS) and paired-pulse TMS measures of short interval intracortical inhibition (SICI), and intracortical facilitation (ICF), were assessed twice on separate visits. Test-retest reliability was evaluated, and changes in TMS measures following transcutaneous auricular vagus nerve stimulation were computed.

**Results:**

Pre-intervention TMS measures showed smaller-than-medium sized differences between CIPN and DN groups. The study found good test-retest reliability for TMS measures, with ICC values between 0.69 and 0.95 for all TMS measures of interest.

**Discussion:**

Overall, TMS measures demonstrated good reliability in this sample of Black and Hispanic/Latino individuals with PN, and these findings provide valuable preliminary data for future studies aimed at establishing the psychometric properties and diagnostic utility of TMS measures in PN.

## Introduction

Peripheral neuropathy (PN) is the most common neurodegenerative disorder, and its prevalence is increasing due to two major causes, the increase in survivorship among cancer patients treated with neurotoxic chemotherapies and the expanding epidemic of diabetes (DiAntonio, 2019; Townsend, 2024). It is estimated that up to 90% of people who receive neurotoxic chemotherapies will develop acute chemotherapy-induced peripheral neuropathy (CIPN) and over 30% develop chronic CIPN (Kerckhove et al., 2017; Seretny et al., 2014). Similarly, PN is believed to affect up to 50% of people with diabetes (DN), causing pain and reducing quality of life (Feldman et al., 2019; Pop-Busui et al., 2017). PN is also known to place greater burden on racial and ethnic minority communities in the United States, particilarly for Black, Hispanic/Latino, and Native American communities (Clayton et al., 2023; Schneider et al., 2015).

Although the precipitating factors that lead to CIPN and DN differ, both are characterized by distal symmetric dysesthesias and paresthesias in glove/stocking distributions. Further, the associated physiological mechanisms believed to underly the perpetuation of neuropathic symptoms are similar for both groups (i.e., neuroinflammation, autonomic dysregulation, and altered central nervous system processing) (Maalmi et al., 2023; Rodwin et al., 2022; Sempere-Bigorra et al., 2021). A review of both human and animal studies suggested that CIPN is partly caused by brain hyperactivity and reduced GABAergic inhibition (Omran et al., 2021). Specifically, neurotoxic chemotherapies have been shown to cause a decrease in GABA in the thalamus (Ferrier et al., 2015), and restoration of GABA levels, or experimentally activating the GABA pathway, reduces CIPN symptoms (Bráz et al., 2015; Kanat et al., 2013; Nagasaka et al., 2017; Nashawi et al., 2016; Xu et al., 2018). Likewise, DN is known to be associated with reduced GABAergic inhibition and increased thalamic excitability (Lee-Kubli et al., 2018; Marshall et al., 2023; Worthington et al., 2021; Zang et al., 2023).

Therefore, non-invasive measurement of GABA in the central nervous system might serve as a mechanistic endpoint for clinical trials for PN, and they could direct the development of non-invasive interventions to maximize GABAergic signaling through endogenous mechanisms (Vucic et al., 2023). Transcranial magnetic stimulation (TMS) is a promising solution, as it can be used to assess corticospinal excitability via the function of GABAergic and glutamatergic and serotonergic pathways in the motor cortex. The clinical diagnostic utility of TMS techniques have been reported across a range of diseases, including neurodegenerative, inflammatory, and lesional brain and spinal disorders (Vucic et al., 2023). Although studies have demonstrated reduced corticospinal-motor plasticity in people with diabetes using TMS (Fried et al., 2017b; Fried et al., 2019; Jannati et al., 2023), these techniques have not been applied to characterize neurophysiological changes associated with CIPN or DN. Prior studies on the role of the central nervous system in PN have largely focused on imaging technologies, with only a few using electroencephalogram (EEG) or electromyography (EMG) for H-reflex assessement, and to our knowledge none have explored the use of TMS measures as diagnostic tools (Omran et al., 2021; Zang et al., 2023).

Emerging evidence suggests that PN symptoms are accompanied by both structural and functional changes in the brain, namely in the pain modulation areas (Cunha et al., 2024). Additionally, both CIPN and DN have been shown to be partly caused by corticospinal hyperactivity and reduced GABAergic inhibition (Bráz et al., 2015; Kanat et al., 2013; Lee-Kubli et al., 2018; Marshall et al., 2023; Nagasaka et al., 2017; Nashawi et al., 2016; Omran et al., 2021; Worthington et al., 2021; Xu et al., 2018; Zang et al., 2023). TMS has been used in neuroscience research for over 40 years, and a wide variety of diagnostic approaches have been developed (Vucic et al., 2023). There are four neurophysiologic applications of TMS that we believe are of particular interest to PN researchers and clinicians given their relative simplicity and potential value:

1. *Motor threshold*

When TMS is applied to the motor cortex (M1) with sufficient stimulation intensity, motor evoked potentials (MEPs) can be recorded from contralateral extremity muscles (Kobayashi and Pascual-Leone, 2003). The lowest intensity required to consistently evoke MEPs in the target muscle is referred to as the motor threshold (MT). While MT is known to be an indicator of short-lasting glutamatergic AMPA transmission in corticospinal neurons (Di Lazzaro et al., 2003; Vucic et al., 2023; Ziemann et al., 1996), it is important to note that MT is also result of the combined excitability of (1) the core of neurons that represent the target muscle in M1, (2) the interneurons projecting onto these neurons, (3) motor neurons in the brainstem and spinal cord, and (4) neuromuscular junctions and muscle (Kobayashi and Pascual-Leone, 2003; Vucic et al., 2023; Ziemann et al., 1996). Thus, MT provides insights on the efficacy of the entire pathway being investigated (e.g., from the presynaptic cortical neurons to the target muscle) (Kobayashi and Pascual-Leone, 2003).

2. *MEPs with unconditioned single pulse TMS (spTMS)*

Cortical excitability is also represented by the amplitude of MEPs elicited by a single suprathreshold (delivered at an intensity above MT) TMS pulse stimulus (Kobayashi and Pascual-Leone, 2003).

3. *Short latency intracortical inhibition (SICI)*

SICI involves stimulating the primary motor cortex (M1) with a subthreshold conditioning-stimulus followed by suprathreshold test-stimulus at inter-stimulus intervals (ISI) of 1-6 ms. This typically results in a decreased MEP amplitude compared to the test-stimulus alone (Vucic et al., 2023), and pharmacological studies have suggested SICI is mediated by inhibitory inter-neuronal circuits acting via GABA-A receptors (Di Lazzaro et al., 2007; Ziemann et al., 2015).

4. *Intracortical facilitation (ICF)*

Using the same parameters as SICI while simply increasing the ISI to 8-30 ms results in the opposite effect: an increased MEP amplitude compared to the test-stimulus alone (Vucic et al., 2023). This technique is termed intracortical facilitation (ICF), and it is an NMDA receptor dependent phenomenon that is modulated by serotonergic neurotransmission (Gerdelat-Mas et al., 2005; Ziemann et al., 1998). Thus, both SICI and ICF examine corticospinal excitability but through different mechanisms.

Given that PN symptoms are accompanied by both structural and functional changes in the brain, non-invasive brain stimulation (NIBS) techniques are promising tools for managing PN and warrant exploration. TMS measures may be used as a probe to test resulting neurophysiological changes with interventions and thus improve understanding of the underlying mechanisms for the effects of these interventions on PN. Before this can be done, the feasibility of conducting these measures, and the psychometric properties and normative data on these measures, must be established in this population.

**FIGURE 1:**
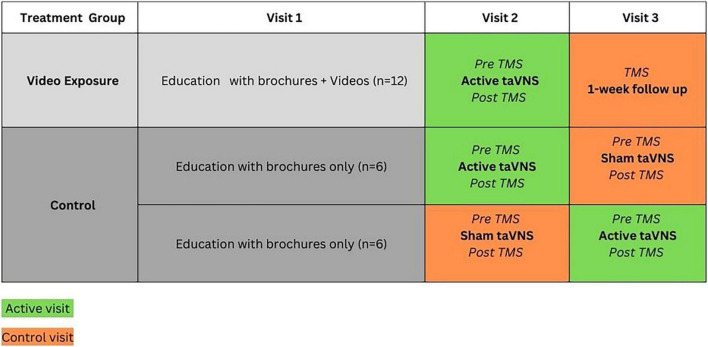
Project overview. NIBS, non-invasive brain stimulation; TaVNS, transcutaneous auricular vagus nerve stimulation. *^a^*Wong et al. (2025). *^b^*Wong et al. (2024).

Further, Black and Hispanic/Latino people have historically been severely underrepresented in TMS research (Peebles et al., 2023), despite experiencing a higher prevalence of CIPN and DN (Clayton et al., 2023; Schneider et al., 2015). It is important to include these communities in this research to ensure that the research reaches all of those who may benefit and to make research findings more generalizable. This study is a component of a larger project aimed to develop and test videos to enhance recruitment and consenting of racial/ethnic minorities in NIBS research (Figure 1). The primary purpose of this study was to provide preliminary and detailed descriptive data on TMS measures in a cohort of Black and Hispanic/Latino patients with chronic PN.

## Materials and methods

**FIGURE 2:**
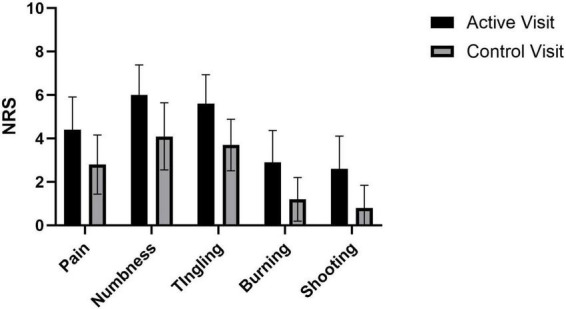
Pilot sham-controlled RCT study design. TMS, transcranial magnetic stimulation assessment.

This study is a secondary aim from a pilot randomized sham-controlled trial (RCT) that was designed to examine the influence of educational videos on transcutaneous auricular vagus nerve stimulation (taVNS) on participant expectations for pain relief with taVNS in Black and Hispanic/Latino people with CIPN (*n* = 17) or DN (*n* = 11). A detailed description of the parent study is provided elsewhere (clinicaltrials.gov #NCT05896202) (clinicaltrials.gov, 2024; Wong et al., 2024). Briefly, participants were randomly assigned to video (intervention) or control groups, and all participants completed three visits (Figure 2): The first visit consisted of ∼90 min of education on both taVNS and TMS, including review of brochures and consent forms (both groups) and 3 short video segments on taVNS for the intervention group; the second visit consisted of a baseline assessment battery, which included TMS measures, followed by a 60-min active taVNS session for the intervention group and randomly assigned active or sham taVNS for the control group, and then the assessment battery was repeated; the 3rd visit consisted of only the assessment battery (control visit) for the intervention group, and the control group were crossed over such that those who received the active taVNS on visit 2 received sham on visit 3 (control visit), and the assessment battery was administered pre and post active or sham taVNS. Pre-intervention TMS measures obtained from the active and control visits (separated by at least 48 h) were used to assess test-retest reliability of TMS measures as there is no evidence that a single session of taVNS induces lasting effects on cortical excitability. This was confirmed by analyzing 95% confidence intervals and effect sizes for the change in measures between visits. All confidence intervals contained 0, and less than medium effect sizes (Cohen’s d values ranged from 0.05 to 0.36) were observed for changes in TMS measures (see Results).

### Participants

Participants were recruited from the University of Miami medical health care system from January to May 2024. Potential participants were identified by medical record and then their respective providers (i.e., oncologist or endocrinologist) informed them about the pilot study during clinical visits. Inclusion criteria included anyone with glove or stocking distribution paresthesia or dysesthesia that developed after receiving neurotoxic chemotherapies, or with a medical diagnosis of DN, and who self-identified as Black or Hispanic/Latino and had a score on the Neuropathic Pain Symptom Inventory (Bouhassira et al., 2004) greater than 10. Exclusion criteria included (1) any unstable medical condition or medical contraindication to moderate physical exertion (e.g., unstable angina or cardiac arrythmia), (2) pregnancy, (3) presence of cognitive impairment or language barrier that impairs full autonomy in the consent process or in the ability to participate in detailed interviews, (4) implants in the head or neck, cochlear implants, or pacemaker, (5) head or neck metastasis or recent ear trauma, (6) history of seizures. Approval was granted by the University of Miami Institutional Review Board, and informed consent was obtained from all participants.

### Procedures

In the first visit, all participants were educated on taVNS and TMS using brochures and the consent form, and the intervention group received video enhanced education on taVNS. For the active (real taVNS) and control (sham taVNS or measurement only) visits, all participants received the same TMS measures at baseline and after real or sham taVNS.

### TMS measures

Participants were seated in an armchair with the head on a headrest throughout the visit. TMS was delivered through a MC-B70 angled focal figure-of-eight shaped magnetic coil connected to a Magpro X100 (MagVenture, Alpharetta, GA). Motor evoked potentials (MEP) were recorded with the PowerLab system (AD Instruments, New Zealand) using surface EMG Ag/AgCl electrodes placed over the right first dorsal interosseous muscle in a belly-tendon montage. The coil position resulting in both visible twitch of the target muscle and the highest peak-to-peak amplitude of the MEP (“hotspot”) was marked using the TMS navigation system (Localite, Germany) to ensure accurate coil positioning throughout the testing. A fixed sequence of TMS measurements were followed:

1. Resting motor threshold (RMT) was defined as the lowest stimulator output intensity that induced MEP peak-to-peak amplitude greater than 50 μV in five out of 10 consecutive trials.
2. A combination of spTMS (at 130% of RMT) and different ppTMS (paired-pulse TMS) conditions were administered in an intermixed, pseudorandom order. For ppTMS, the conditioning- and test stimuli were set to 70 and 130% RMT, respectively. A range of interstimulus intervals (ISIs) were tested, spanning SICI and ICF (1, 2, 3, 7, 10, and 15 ms) to create a ppTMS curve. By covering a range of ISIs our intent was to minimize the risk of missing the peak ISI for a given individual. The average of the 10 trials was used to define the amplitude of the peak-to-peak MEP for each condition, and the mean of all trials at ISIs 1, 2, and 3 (30 pulses) was used for SICI, and the mean of all trials at ISIs 7, 10, and 15 (30 pulses) was used for ICF.

RMT is presented as a percentage of machine maximum output (%MMO), and spTMS is presented in millivolts (mV). SICI and ICF values are presented as the relative percentage of mean paired pulse MEP values to mean spTMS MEP values, minus 100 [(conditioned response/unconditioned response) × 100%)-100], such that negative values represent relative inhibition, and positive values represent relative facilitation.

### Neuropathic pain assessment

The Neuropathic Pain Symptom Inventory (NPSI) (Bouhassira et al., 2004) was administered as part of the screening process. The NPSI is one of the most widely used tools for characterizing neuropathic pain symptom severity (Bouhassira et al., 2004; Haanpää et al., 2011), and it assess dimensions of neuropathic pain (burning spontaneous pain, pressing spontaneous pain, paroxysmal pain, evoked pain, and paresthesia/dysesthesia) over the last week. NPSI total score ranges from 0 to 100, with higher scores indicating worse NP severity. Additionally, at each visit participants were asked to rate the severity of their symptoms at the immediate moment with 0-10 numeric rating scales for pain, numbness, tingling, burning, and shooting/electric shocks; this was asked both before and after taVNS stimulation.

### Analyses

The project was designed and powered for qualitative analysis (Wong et al., 2024) rather than to detect differences based on either groups or timepoints; however, the sample size provided 80% power (α = 0.05) to detect a medium-to-large within-subject effect (d ≥ 0.70) or a large correlation (*r* ≥ 0.52). Therefore, we focus on descriptive statistics (i.e., means, medians, standard deviations, and confidence intervals), and we provide tests of significance, with reported effect sizes, to serve as a useful guide for future studies (see Table 1).

**TABLE 1:** Descriptive statistics for TMS measures.

	RMT (% MMO)	SP (mV)	SICI (% of SP)	ICF (% of SP)
Active visit (*n* = 23)	66.7/70.0 (13.1)	1.5/0.9 (1.4)	–35.5/–40.9 (33.0)	17.7/10.1 (33.7)
Control visit (*n* = 19)	66.3/70.0 (12.3)	1.4/0.9 (1.1)	–41.4/–51.4 (28.3)	26.8/20.0 (41.6)
Cohen’s d (*p*-value*)	0.19 (0.31)	0.00 (0.94)	0.27 (0.15)	0.24 (0.30)
ICC (95% CI)	0.95 (0.86-0.98)	0.83 (0.55-0.93)	0.94 (0.84-0.98)	0.69 (0.17-0.88)
**Diagnosis**				
CIPN (*n* = 14)	69.9/71.0 (11.3)	1.7/1.0 (1.6)	–33.3/–37.7 (30.8)	15.2/6.8 (39.1)
DN (*n* = 9)	61.7/62.0 (14.8)	1.1/0.7 (0.9)	–39.3/–55.9 (37.6)	21.6/15.3 (24.7)
Cohen’s d (*p*-value**)	0.64 (0.16)	0.43 (0.42)	0.19 (0.60)	0.19 (0.31)
**Racial/ethnic**				
Hispanic (*n* = 13)	64.8/70.0 (14.2)	1.3/0.9 (1.4)	–32.9/–38.5 (32.1)	18.4/10.1 (39.3)
Black (*n* = 10)	69.0/70.0 (11.7)	1.7/1.0 (1.4)	–38.8/–53.9 (35.4)	16.9/10.5 (26.9)
Cohen’s d (*p*-value^**^)	0.31 (0.69)	0.22 (0.52)	0.18 (0.78)	0.04 (0.93)

Mean/median (standard deviation) values; RMT, resting motor threshold; MMO, machine maximum output; SP, single pulse at 130% of resting motor threshold; SICI, short intracortical inhibition; ICF, intracortical facilitation; ICC, intraclass correlation coefficient; CI, confidence interval; CIPN, chemotherapy induced peripheral neuropathy; DN, diabetic neuropathy;

*p-values based on Wilcoxon Signed Ranks Test;

**p-values based on Mann-Whitney *U*-test.

Correlations between TMS and measures and neuropathic symptoms were assessed using Spearman’s rho. As differences between active and sham taVNS sessions were smaller than medium effects, test-retest was performed instead on visits in the order they were performed (first-test, second-retest). ICC analysis was conducted using an alpha two-way mixed model for absolute agreement to assess the test-retest reliability of TMS measures, and the results are reported with 95% confidence intervals. All statistical analyses were conducted using Statistical Package for the Social Sciences (SPSS) v28 (IBM Corp., Armonk, NY) and figures were rendered using GraphPad Prism v9.3.1 (GraphPad Software, La Jolla, CA).

## Results

**FIGURE 3:**
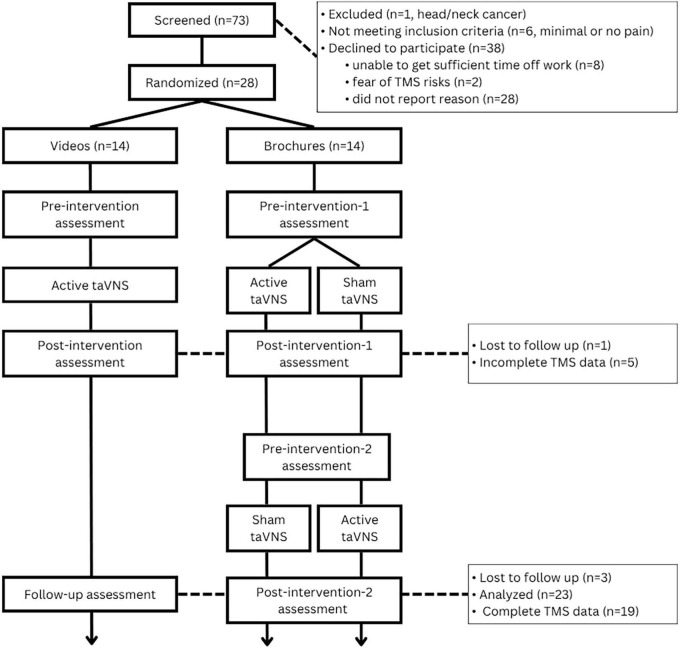
CONSORT diagram.

**FIGURE 4:**
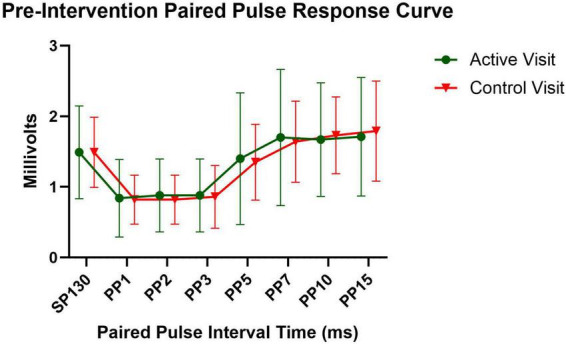
Pre-intervention symptoms at active and control visits. Mean with 95% Confidence Interval; NRS, numeric rating scale.

Twenty-eight participants were enrolled (17 with CIPN and 11 with DN); however, only 24 completed both active and control visits, with 23 included in partial data analysis and 19 with complete TMS data (Figure 3). Five participants were missing TMS data (21%) because their RMT exceeded 77% of the machine’s maximum output, and therefore a stimulus intensity of 130% of RMT could not be achieved for paired pulse assessments. Demographic and visit 1 pain information for the participants can be found in Table 2. Briefly, 10 identified as Black non-Hispanic/Latino, and 13 identified as Hispanic/Latino (11 White and 2 Black). All participants had a medical diagnosis of PN from a provider, with 17 (74%) reporting symptoms > 1 year, 4 (17%) between 6 months to a year, and 2 reporting onset of symptoms between 3 and 6 months prior. Participants with DN had slightly higher symptoms on average than the participants with CIPN, but there were wide ranging scores across both groups and no meaningful differences between them (all *p*-values > 0.14). Pre-intervention scores at active and control visits can be found in Figure 4.

**TABLE 2:** Demographic and pain characteristics.

	CIPN (n = 14)	DN (n = 9)	Total sample (n = 23)	P-value
Age*	60.4 (9.2)	54.4 (8.2)	58.0 (9.1)	0.18
Gender (% female)	78.6	66.7	73.9	0.53
Using pain medication (%Yes)	85.7	55.6	73.9	0.11
• Duloxetine (n)	0	1	1	0.20
• Gabapentin (n)	10	5	15	0.44
• Opioids (n)	0	1	1	0.20
Pain* (0–10)	6.9 (2.5)	8.3 (1.1)	7.5 (2.2)	0.13
Numbness* (0–10)	7.8 (1.9)	8.6 (1.3)	8.1 (1.7)	0.35
Tingling* (0–10)	7.8 (2.0)	8.0 (1.6)	7.9 (1.8)	0.90
Burning* (0–10)	6.6 (3.2)	5.6 (3.9)	6.2 (3.4)	0.57
Shooting* (0–10)	5.9 (3.3)	7.7 (3.3)	6.6 (3.3)	0.13
NPSI score* (0–100)	57.7 (20.1)	68.7 (19.2)	62.0 (20.1)	0.12
EXPECT score* (0–10)	8.0 (2.0)	7.5 (1.2)	7.8 (1.7)	0.21

*Mean (standard deviation).

### Baseline TMS measure descriptives

The differences in baseline TMS measures pre-intervention between participants with CIPN and those with DN were small to moderate (Table 1), with median values for the entire sample of 70.0 for RMT, 0.9 mV for SP, –40.9% for SICI, and 10.1% for ICF. Additionally, there were small effect sizes for differences in pre-intervention values between active and control visits, with mean differences of –1.1 for RMT, 0.0 mV for SP, 4% for SICI, and –9% for ICF (Table 1).

### Association of TMS measures with neuropathic symptoms

Preintervention TMS measures were not significantly associated with preintervention neuropathic symptoms, with *r*-values ranging from 0.00 to 0.43 (Table 3). SICI demonstrated the most consistent relationship with symptoms, with decreased SICI (higher values) associated with higher shooting/electric shock symptoms (*r* = 0.34, *p* = 0.12) and feeling of pressure (0.43, *p* = 0.05).

**TABLE 3:** Association of pre-intervention TMS measures with symptom severity.

	RMT	spTMS	SICI	ICF	Burning	Shooting	Tingling	Numbness	Pressure	Pain
RMT	1.00	–0.13 (0.56)	–0.21 (0.33)	0.07 (0.75)	–0.16 (0.47)	0.13 (0.55)	0.20 (0.35)	0.05 (0.83)	–0.11 (0.65)	0.12 (0.58)
spTMS		1.00	–0.30 (0.17)	–0.13 (0.57)	–0.00 (0.99)	–0.09 (0.69)	–0.07 (0.76)	–0.23 (0.28)	–0.24 (0.29)	0.02 (0.92)
SICI			1.00	0.25 (0.25)	0.17 (0.43)	0.34 (0.12)	0.26 (0.23)	0.25 (0.25)	0.43 (0.05)	0.28 (0.19)
ICF				1.00	–0.05 (0.84)	0.17 (0.44)	0.01 (0.97)	0.04 (0.86)	0.03 (0.91)	0.16 (0.46)
Burning					1.00	0.61^**^ (0.00)	0.57^**^ (0.01)	0.61^**^ (0.00)	0.76^**^ (0.00)	0.61^**^ (0.00)
Shooting						1.00	0.73^**^ (0.00)	0.50* (0.02)	0.80^**^ (0.00)	0.77^**^ (0.00)
Tingling							1.00	0.70^**^ (0.00)	0.64^**^ (0.00)	0.82^**^ (0.00)
Numbness								1.00	0.62^**^ (0.00)	0.62^**^ (0.00)
Pressure									1.00	0.79^**^ (0.00)
Pain										1.00

RMT, resting motor threshold; spTMS, unconditioned MEP amplitude; SICI, short interval intracortical inhibition; ICF, intracortical facilitation.

^**^Correlation is significant at the 0.01 level (2-tailed).

*Correlation is significant at the 0.05 level (2-tailed).

### Test-retest reliability of TMS measures

**FIGURE 5:**
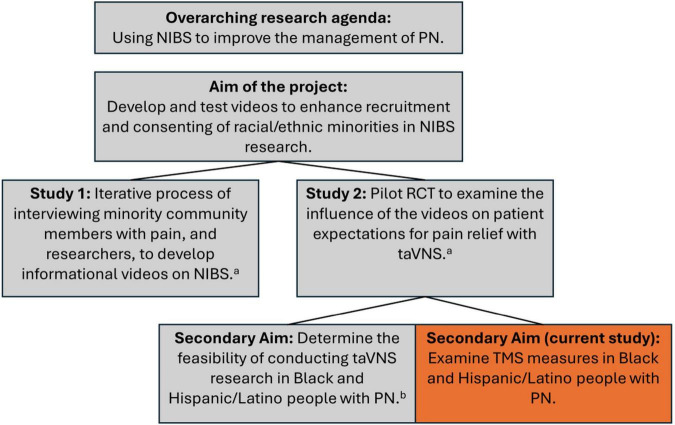
Pre-intervention paired pulse response curve. Mean with 95% Confidence Interval. sp130, single pulse at 130% of resting motor threshold; PP, paired pulse followed by the interstimulus interval value.

The median number of days between visits was 5 (with a mean of 6.2 and a range of 2-26 days). Mean NRS scores for all symptoms decreased slightly between visits (mean change scores ranged from –1.6 to –0.8), and there was no difference between the participants with CIPN and those with DN (*p*-values > 0.10). For the entire cohort group, there was excellent to moderate test-retest reliability of TMS measures of interest (ICCs ranging from 0.69 to 0.95), with RMT and SICI demonstrating the highest ICC values (Table 2). ICC values for individual paired pulse intervals ranged from 0.69 to 0.86 indicating fair to good stability, with higher values for the ISIs associated with inhibition (i.e., ISIs 1-3, Figure 5).

## Discussion

In this study, we provide the first detailed description of TMS measures in people with painful PN associated with either neurotoxic chemotherapy exposure or diabetes. Importantly, the study sample consisted entirely of people who self-identified as Black or Hispanic/Latino, which are communities that are historically underrepresented in TMS research (Peebles et al., 2023). Although hair type and style has been reported as a key barrier to conducting TMS research in Black communities (Peebles et al., 2023), this was likely only a contributing factor for two of the five participants that we could not collect TMS data on. For these participants, the volume of hair prevented close contact of the coil with the scalp, and it is known that RMT increases linearly with the coil-cortex distance (Stokes et al., 2007). Thus, the high volume of hair between the coil and scalp likely elevated the required stimulus to achieve RMT beyond 77% of the maximum machine output, which was the ceiling for being able to complete the paired pulse protocol. However, three of the five participants with missing data had no hair or low hair volume, and we were still unable to achieve RMT within 77% of the maximum machine output.

### Association of TMS measures with neuropathic symptoms

Unsurprisingly, there was no evidence for strong associations between pre-intervention TMS measures with neuropathic symptom severity. Pain and neuropathic symptoms are complex phenomena, and no single physiological marker has yet proven to have consistent and strong diagnostic or prognostic value for these symptoms (Bönhof et al., 2019; Bouhassira and Attal, 2023; Colvin, 2019). SICI is a promising marker for pathways involved in neuropathic pain, and although differences have been observed between healthy controls and people with chronic pain (Chiang et al., 2022; Snow et al., 2022), it may be more likely that for some patients, changes in their pain are associated with changes in their corticospinal (i.e., intra vs. inter patient analyses).

### Test-retest reliability

The test-retest reliability was excellent to moderate for all TMS measures. Importantly, SICI had excellent reliability in this cohort of people with PN. The International Federation of Clinical Neurophysiology recently published a comprehensive update on the clinical diagnostic utility of TMS (Vucic et al., 2023), and they concluded that of the TMS techniques, a reduction in SICI was most consistently associated with chronic pain, and therefore SICI might be used as a biomarker to select candidates for analgesic cortical neuromodulation (Vucic et al., 2023). This study’s findings of high test-retest reliability, and a trend towards significance for association with neuropathic symptoms, support the potential for using SICI as a biomarker.

The high heterogeneity in TMS protocols across studies limits our ability to compare the findings from this study. However, RMT and SICI have been established as a stable measures with good reliability in healthy people over the age of 50 (Cueva et al., 2016; Kimiskidis et al., 2004) and in people with diabetes (Fried et al., 2017a), and we found the same to be true in people with PN. One study found median values of –50% for SICI and 40% for ICF in healthy adults over 50 (Cueva et al., 2016), and another study on patients with painful diabetic neuropathy found baseline SICI values between 80 and 85% (Thakkar et al., 2023). The cohort in this study may have diminished capacity for both corticospinal inhibition and facilitation based on the findings. However, this cohort had high symptom burden which may have influenced the results. Additionally, there were methodological differences that may have contributed to the differences in findings between this study and the previous studies discussed. Specifically, in this study we used the mean of 30 trials [10 trials at 3 different interstimulus intervals (1, 2, and 3 ms)] to determine SICI, whereas the other studies used the mean of 4 trials at a single interstimulus interval (2 or 4 ms). We also observed high inter-individual variability regarding which single ISI (i.e., 1, 2, or 3 ms) elicited the greatest inhibitory response in each participant. Thus, the methodology used in this study (i.e., averaging across multiple ISIs), and the higher number of trials used to establish SICI may be considered a strength, as this methodology may provide a more stable and generalizable measure than using fewer trials with a single ISI. There is wide heterogeneity of methods in TMS research, and research is needed to determine best practices for TMS measurement.

### Limitations

It is important to note that an intervention was provided between TMS measurements which may have affected the validity of the test-retest analysis. Although it is possible that there was a lasting effect from taVNS, the combination of the small effect size of differences between active and sham taVNS sessions, and the high observed ICC values, supports the assumption that a single session of taVNS did not have lasting effects on these TMS measures. The findings of this study should also be viewed as preliminary and interpreted with caution given the small sample size. Despite the limited generalizability, the fact that our entire sample consisted of Black and Hispanic/Latino people with PN adds value to the findings, as these communities have thus far been underrepresented in TMS research (Peebles et al., 2023). Future studies are needed with diverse samples to determine the psychometric properties of these measures of people with PN.

## Conclusion

In this sample of Black and Hispanic/Latino people with PN, TMS measures were found to have good test-retest reliability. Additionally, we provided descriptive data on TMS measures that can be used for planning future studies to conclusively determine the psychometric properties and diagnostic utility of TMS measures for PN. The findings of this study suggest that TMS measures, and SICI in particular, may be promising tools for examining neurophysiological changes associated with PN and its treatment.

## Data Availability

The raw data supporting the conclusions of this article will be made available by the authors, without undue reservation.
